# Three-dimensional geometric morphometric analysis reveals ethnic dimorphism in the shape of the femur

**DOI:** 10.1186/s40634-017-0088-2

**Published:** 2017-05-02

**Authors:** Etienne Cavaignac, Ke Li, Marie Faruch, Frederic Savall, Philippe Chiron, W. Huang, Norbert Telmon

**Affiliations:** 1 Laboratoire AMIS, UMR 5288 CNRS, Université Paul Sabatier, 37 allée Jules Guesdes, 31000 Toulouse, France; 2 Institut de l’appareil locomoteur, Hôpital Pierre-Paul Riquet, Toulouse, CHU France; 3 Department of Orthopedic Surgery, The First Affiliated Hospital of Chongqing Medical University, Chongqing, China

**Keywords:** Distal femur dimorphism, Principal component analysis, Procrustes analysis, Geometric morphometric analysis, Biological anthropology

## Abstract

**Background:**

Ethnic dimorphism in the distal femur has never been studied in a three-dimensional analysis focused on shape instead of size. Yet, this dimorphism has direct implications in orthopedic surgery and in anthropology. The goal of this study was to show that differences in distal femur shape related to ethnic dimorphism could be identified, visualized, and quantified using 3D geometric morphometric analysis.

**Methods:**

CT scans of the distal femur were taken from 482 patients who were free of any bone-related pathology: 240 patients were European (E) and 242 were Asian (A). Ten osteometric landmarks based on standard bone landmarks used in anthropometry were placed on these scans. Geometric morphometric analysis, principal component analysis (PCA), canonical variates analysis (CVA), and other discriminant analyses (Goodall’s F-test and Mahalanobis distance) were performed. A cross-validation analysis was carried out to determine the percentage of cases in which the ethnicity was correctly estimated.

**Results:**

The shape of the E and A distal femur differed significantly (Goodall’s F = 94.43, *P* < 0.001 and Mahalanobis D2 distance = 1.85, *P* < 0.001). PCA identified a difference in distal femur shape between A and E. The CVA revealed that correct ethnicity was assigned in 82% of cases and the cross-validation revealed a 75% rate of correct ethnic group estimation.

**Conclusion:**

The distal femur exhibits ethnic dimorphism. 3D geometric morphometric analysis made it possible to demonstrate these differences. The large number of subjects studied has helped modernize the references for certain bone measurements, with direct implication for orthopedic surgery and anthropology.

## Background

Ethnic diversity is always an important element that may affect anthropometric data. It has shown that the anatomy of the distal femur varies by ethnic group (Barrier et al. 2009; Bellemans et al. 2010; Bilfeld et al. 2012; Bilfeld et al. 2013; Bookstein 1978; Cavaignac et al. 2016; Cheng et al. 2009; Dai et al. 2014; Elewa 2010; Gonzalez et al. 2009; Ho et al. 2006). These comparisons were based on metric measurements between distinct points on the femur, but not true three-dimensional (3D) analysis (Cheng et al. 2009; Ho et al. 2006). However, these metric methods suffer from analysis bias related to inter- and intra-observer errors, rater experience, standardization challenges and problems related to statistical analysis (Gonzalez et al. 2009).

Geometric morphometric analysis is a useful tool that allows quantification of morphological features. The primary advantage of geometric morphometric analysis over traditional morphological tools is that it uses powerful multivariate statistics tools to investigate morphological variations in the anatomical context of the structure studied (Bilfeld et al. 2012). It provides valuable visual information that can be used to study differences between skeletal features. It was developed to quantify the shape of rigid structures consisting of curves and bulges that are not easy to interpret using traditional metric methods (Bookstein 1978). Geometric morphometric analysis has been used since the 1980s, but has only become popular in anthropology recently (Pretorius et al. 2006). This method can be used to perform diachronic and interethnic comparisons (Cavaignac et al. 2016). This method allows the shape of two or more objects to be compared while disregarding the volume of these objects (Bilfeld et al. 2012). Since the size is normalized, the analysis can focus on the shape.

To the best of our knowledge, this method has not been used to analyze ethnic dimorphism in the distal femur. Measurement of this dimorphism has direct implication for orthopedics. The shape of the distal femur has a direct impact on the design of total knee replacement implants. Kim et al. recently published a systematic review that looked into the anatomical differences in the knee of patients of various races (Kim et al. 2017). All the comparisons reviewed by Kim et al used classic osteometric methods. Although some of the osteometric analyses were done in various planes in space, they were not truly three-dimensional. In addition, these classic osteometric parameters are affected by the size of the objects being compared. It is widely known that the anatomical profiles of Asian knees are smaller and narrower than those of Caucasian (Yue et al. 2011). However, we were not interested in analyzing size variations, as size variations can be compensated for by using a different size implant. Instead, we were interested in shape differences, which may bring into question the design of the implant itself. Geometric morphometric analysis studies the shape by disregarding size-related effects.

We hypothesized that 3D geometric morphometric analysis of the distal femur would reveal differences between ethnic groups. The primary goal of this study was to show that differences in distal femur shape related to ethnic dimorphism could be identified, visualized, and quantified using 3D geometric morphometric analysis. The secondary goal was to quantify the differences observed in the 3D anatomy of the distal femur relative to ethnic group and sex.

## Methods

This was a retrospective descriptive analytical study. The research ethics committee at our respective healthcare facilities approved this study (No. 01-0415 and No. 2016-94).

### Materials

#### Study population

The analysis was carried out on the CT images of 482 distal femurs. Only scans showing the entire distal femur (tip of femoral groove to most distal aspect of femur) were retained. Any CT scans with signs of pathology or osteoarthritis in the distal femur were excluded. The included CT scans had been performed to assess leg vasculature (CT angiogram). Between June 1, 2014 and December 31, 2014, 482 CT scans of the distal femur met our inclusion criteria: 240 patients were European (E) (from southwest France) and 242 were Asian (A) (Huan from Chongqing, China). There were 228 women (122 Asian and 106 European and 254 men (137 Asian and 117 European). The average age was 55.3 ± 15.2 years. The right side was analyzed 235 times and the left side 247 times. The two groups were comparable in terms of their demographics (Table 1).

**Table 1:** Mean age of the various subgroups relative to sex, side and ethnicity

		Age
Sex	Male (*n* = 254)	55.24 ± 15.20
	Female (*n* = 228)	55.45 ± 16.47
Side	Right (*n* = 235)	55.14 ± 6.24
	Left (*n* = 247)	55.53 ± 15.59
Ethnicity	European (*n* = 240)	56.47 ± 14.85
	Asian (*n* = 242)	54.22 ± 16.80

Comparisons were performed with Student’s *t*-test – *P* > 0.05 for all comparisons

The CT scans were taken on a Sensation 16 (120 kV, 80 mA; light speed 16) Scanner (Siemens, Erlangen, Germany) with 16*1.5 mm collimation. The image matrix was 512*512 pixels. A bone filter and a soft tissue filter were used. The scanning protocol was carried out to acquire axial 2-mm reconstructions every 1 mm.

The CT scans were saved as digital imaging and communications in medicine (DICOM) files and then processed with Amira® software (version 4.1.1, FEI Visualization Sciences Group, Bordeaux, France).

### Methods

#### 3D morphological analysis

**Fig. 1:**
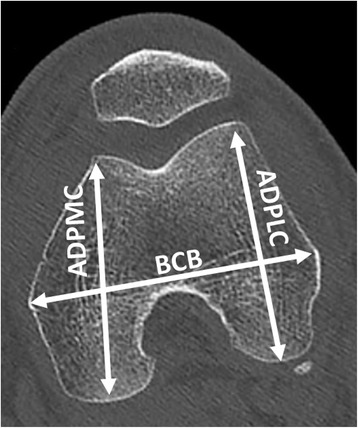
Osteometric data used to measure the plausibility of the study’s methodology. BCB: bicondylar breadth, distance between the two epicondyles, APDMC: anterior posterior diameter of the medial condyle, which is largest anteroposterior dimension of the medial condyle and APDLC: anterior posterior diameter of the lateral condyle, which is largest anteroposterior dimension of the lateral condyle

Ten osteometric landmarks were defined based on standard bone landmarks used in anthropometry (Bellemans et al. 2010). These landmarks were located at the 1) medial epicondyle, 2) most dorsal point on medial condyle, 3) top of intercondylar notch, 4) most dorsal point on lateral condyle, 5) lateral epicondyle, 6) most ventral point on lateral edge of trochlear groove, 7) most distal point at bottom of trochlear groove, 8) most ventral point on medial edge of trochlear groove, 9) most distal point on medial condyle, and 10) most distal point on lateral condyle. By using points typically associated with osteometric techniques, comparisons could be made with published studies to determine the plausibility of our results. Three metric parameters were measured: the bicondylar breadth (BCB), which is the distance between the two epicondyles (Slaus et al. 2003), the anterior posterior diameter of the medial condyle (APDMC), which is the largest anteroposterior dimension of the medial condyle, (Srivastava et al. 2012) and the anterior posterior diameter of the lateral condyle (APDLC), which is the largest anteroposterior dimension of the lateral condyle (Srivastava et al. 2012) (Fig. 1). The landmarks were positioned using 3D in vivo imaging software (Amira®) using the volume rendering technique (VRT) mode and the multi-planar reconstruction (MPR) mode. Once these landmarks had been defined, the coordinates of each landmark in space (x,y,z) were recorded.

During the scan, the subject was placed in a supine position with their knee in a relaxed and extended position. Axial slices perpendicular to the femoral long axis in which the epicondyles were the most prominent were used to place points 1–8. Oblique slices were created by resampling the image stack in order to be orthogonal to the axial plane; points 9 and 10 were placed on these images.

#### Reliability studies

The analyzed data were taken from the same database and analyzed twice on separate occasions by two observers. This made it possible to calculate the intra- and inter-observer variability for each landmark. The percentage error for each landmark was calculated, as described previously (Table 2). The results were deemed acceptable if this error was less than 5%.

**Table 2:** Anatomical description of the various landmarks used, with the intra- and inter-observer variability for each. The error is given as a percentage

Landmark	Location	Intra-observer Variability	Inter-observer Variability
1	Medial epicondyle	1.77	1.82
2	Most dorsal point on medial condyle	1.45	1.46
3	Top of intercondylar notch	1.52	1.60
4	Most dorsal point on lateral condyle	1.77	1.89
5	Lateral epicondyle	1.68	1.64
6	Most outside point on trochlear groove	1.59	1.62
7	Most distal point at bottom of trochlear groove	1.66	1.69
8	Most ventral point on margin of trochlear groove	1.62	1.72
9	Most distal point on medial condyle	1.73	1.69
10	Most distal point on lateral condyle	1.62	1.52

#### Procrustes analysis

**Fig. 2:**
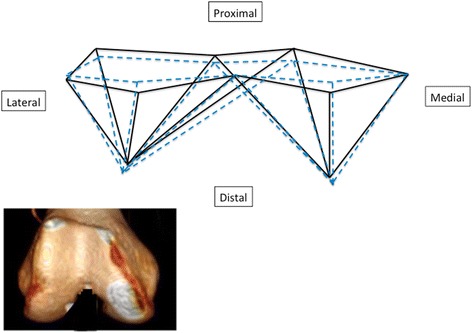
Shape variation based on ethnicity. A 3D reconstruction is shown to make it easier to understand the data (Asian in *blue*, European in *black*)

All morphometric geometric analyses were carried out with Morpho J software and R 2.2.0 software. The chosen landmarks made it possible to characterize the shape of the distal femur (Fig. 2). The first step consisted of a generalized Procrustes analysis (GPA) (Klingenberg 2002). With GPA, size effects related to isometry were removed, but allometric size differences were retained and visible. This strategy expresses the results in graphical format by showing the average shape of the subgroups of interest.

### Statistical analysis

The descriptive analysis consisted of calculating the mean, median and standard deviation values for each subgroup. Normal distribution of continuous variables was verified using the Shapiro-Wilk test and homogeneity of variances was determined using Fisher’s F-test and Levene’s test to ensure the assumptions were met for use of parametric tests. Comparisons of subgroup demographics were performed with Student’s *t*-test. The length variables (BCB, APDLC and APDMC) in the various subgroups were compared using an analysis of variance (ANOVA).

The landmark coordinates were analyzed using principal component analysis (PCA) (Miriam 2004) and canonical variate analysis (CVA) to identify shape trends in the various subgroups (Bilfeld et al. 2013).

To determine if the difference between shapes was statistically significant, a *P*-value was also calculated using Goodall’s F-test and Mahalanobis D2 matrices (Ozer & Katayama 2008; Pretorius et al. 2006). Goodall’s F-test allows testing for overall shape differences between groups while taking all sample variables into account.

A discriminant analysis with leave-one-out cross-validation was performed to determine the percentage of cases in which the ethnic group was correctly estimated. Pearson’s Chi-square test was also performed to compare the percentages of correct ethnic group classification in order to determine if this analysis was statistically significant (Elewa 2010).

## Results

### Reliability analysis

The percentage errors for the intra- and inter-observer comparisons for all the landmarks are given in the Appendix. None exceeded 2% (Table 2).

### Ethnic dimorphism

The mean BCB value was greater in Europeans (80.5 ± 6.5 mm) than Asians (76.3 ± 5.2) (*P* < 0.001). Similar results were found for the APDMC (E: 63.7 ± 5.1, A: 58.5 ± 4.2; *P* < 0.005) and the APDLC (E: 62.8 ± 4.9, A: 58.9 ± 3.8, *P* < 0.001) (Table 3).

**Table 3:** Mean values (± standard deviation) of the osteometric data for each subgroup based on ethnicity and sex

	Asian		European	
BCB	76.3 ± 5.2		80.5 ± 6.5	
APDMC	58.5 ± 4.2		63.7 ± 5.1	
APDLC	58.9 ± 3.8		62.8 ± 4.9	
	ASF	ASM	EUF	EUM
BCB	72.1 ± 3.2	80.0 ± 3.6	75.5 ± 3.7	85.0 ± 4.9
APDMC	55.8 ± 3.3	60.9 ± 3.3	60.3 ± 4.0	66.7 ± 4.2
APDLC	56.9 ± 3	60.7 ± 3.6	60.2 ± 3.9	65.2 ± 4.4

Comparisons were performed with an ANOVA – *P* < 0.001 for all comparisons. *ASF* Asian Female, *ASM* Asian Male, *EUF* European Female and *EUM* European Male

**Fig. 3:**
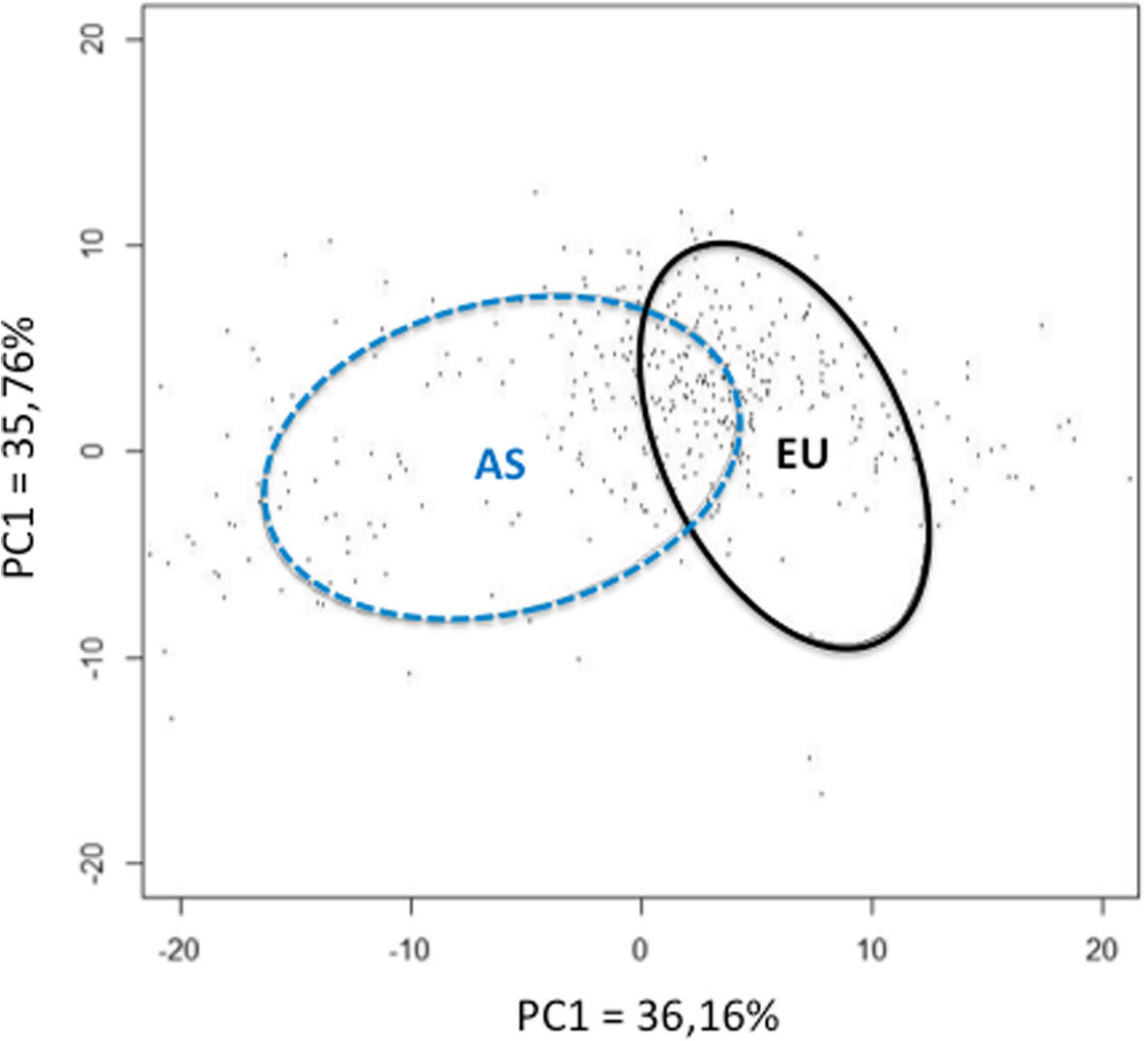
PCA obtained for the shape of the distal femur based on ethnicity. The ellipses correspond to 68% confidence intervals (Asian (AS) in *blue*, European (EU) in *black*)

The shape of the E and A distal femur differed significantly (Fig. 2) (Goodall’s F = 94.43, *P* < 0.001 and Mahalanobis D2 distance = 1.85, *P* < 0.001). For the same femur size, Asian femurs are significantly longer in the frontal plane, i.e. the distance between the axial plane containing the epicondyles and the two most distal points on the condyles is greater in the Asian group. In the axial plane through the epicondyles, Asian femurs are shorter along the anteroposterior axis than European femurs, while the mediolateral distance is the same. The graphical PCA representation that provided the best discrimination in terms of ethnic dimorphism was PC1 against PC2. PCA identified a difference in distal femur shape between A and E; PC1 and PC2 accounted for 71.9% of the variance measured (Fig. 3). CVA revealed that the correct ethnic group was assigned in 82% of cases and the cross-validation revealed a 75% rate of correct ethnic estimation (Table 4).

**Table 4:** Results of the CVA and cross-validation for the ethnic estimation

	Original CVA			Cross-Validated		
	Correctly assigned	Incorrectly assigned	% Correctly assigned	Correctly assigned	Incorrectly assigned	% Correctly assigned
A	203	39	83	187	53	77
E	195	45	81	179	63	73
Total	398	84	82	366	116	75

### Ethnic and sex differences

**Fig. 4:**
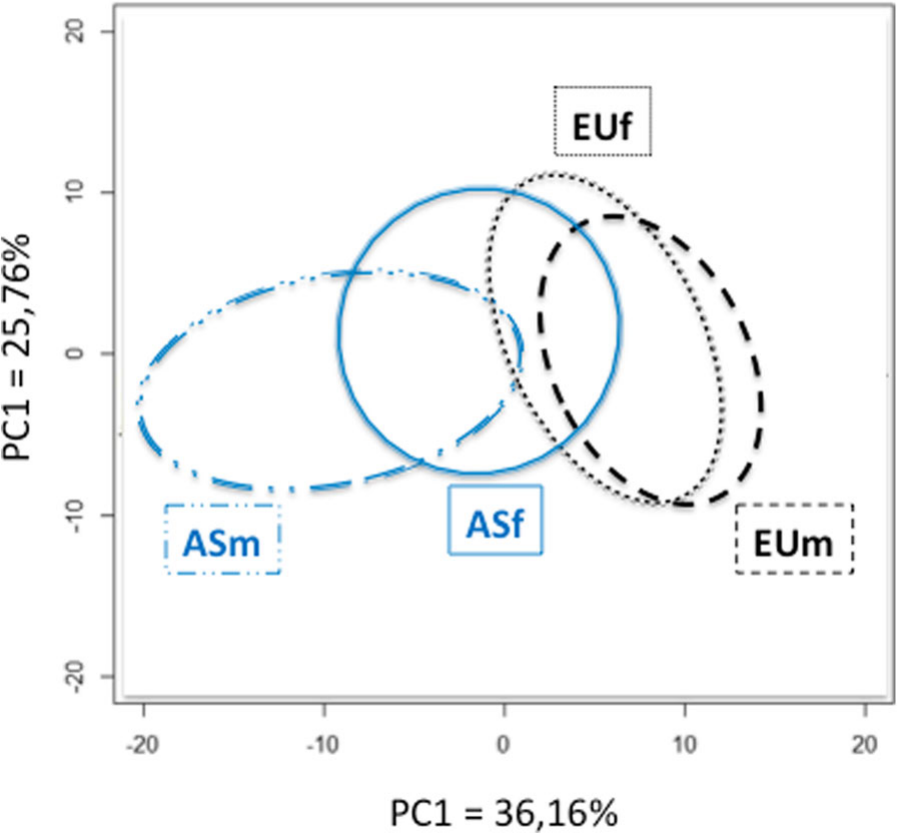
PCA obtained for the shape of the distal femur based on sex and ethnicity. The ellipses correspond to 68% confidence intervals (Asian males (ASM) in *blue dotted line*, Asian females (ASF) in *blue* continuous line, European males (EUM) in *black dashed line*, European females (EUF) in *black* *dotted line*)

The osteometric analysis (BCB, APDMC and APDLC) revealed significant differences between subgroups of subjects (Table 3). The PCA based on ethnicity and sex is shown in Fig. 4; PC1 and PC2 accounted for 61.9% of the variance measured.

## Discussion

Our hypothesis is confirmed: 3D geometric morphometric analysis of the distal femur revealed differences between ethnic groups (Figs. 2 and 3). There are ethnic and ethnic–sexual dimorphisms in the distal femur. All the comparisons performed in this study were statistically significant. The 3D analysis and osteometric data revealed dimorphisms related to ethnicity. Moreover, the PCA analysis (Figs. 3 and 4) and comparative analysis of metric data (Table 3) revealed dimorphisms related to ethnicity, but also sex and ethnicity. The greatest dimorphism was found between Asian men and European men (Fig. 3).

To our knowledge, this is the first study comparing the 3D anatomy of the distal femur between two ethnic groups. We are the first group to show differences in distal femur shape that are independent of the difference in size. It is well-known that the anatomical profiles of Asian knees are smaller and narrower than those of Caucasian knees (Yue et al. 2011). However, in our study, we analyzed the differences in shape, not size. We performed a true 3D analysis because the location of each landmark was analyzed relative to the others. This differs from the analysis of two osteometric data points in two planes in space that is often used for comparisons between ethnic groups (Kim et al. 2017).

Geometric morphological analysis effectively minimizes differences related to general somatotype and keeps only the shape differences. Bellemans et al. (Bellemans et al. 2010) have shown that differences in femur shape were related to an individual’s sex and somatotype. Carter and Heath refined it into three somatotypes: endomorph, mesomorph, ectomorph (Sheldon 1950). Skeletal structure and body composition are used to classify individuals into these three groups. Osteometric analysis helps to assign ethnicity, but is subject to the somatotype effect. Geometric morphometric analysis discounts somatotype-related differences, reducing the accuracy of this analysis. Osteometric analysis is subject to two variables (ethnicity and somatotype), while geometric morphometric analysis is subject to only one variable (ethnicity).

One of the main objectives of physical anthropology is to estimate a person’s sex and ethnicity in the forensic or anthropology context (Slaus et al. 2003). Most of the postcranial bones have been used to determine the sex of human remains through various statistical models (Kim et al. 2013). The femur is the longest bone and it is often well preserved (King et al. 1998). But anthropologists must have different algorithms in their diagnostic arsenal for cases where the skeletons are fragmented or when specific populations are analyzed (Iscan & Shihai 1995). The large number of subjects (*n* = 482) included in our study provides osteometric references related to sexual dimorphism in a modern population. Determining ethnicity based on a bone fragment could improve identification of a specimen, particularly when it is not fully intact. This method made it possible to correctly assign ethnicity in 82% of subjects (original CVA) (Table 4). But this is not sufficient to allow the ethnic origin of a specimen to be determined without a doubt. Anthropologists have different algorithms in their diagnostic arsenal for cases where the skeletons are fragmented or when specific populations are analyzed (Mall et al. 2000; Ozer & Katayama 2008; Purkait & Chandra 2004; Slaus et al. 2003; Srivastava et al. 2012; Trancho et al. 1997). This data may be used as a current reference when virtual or in vivo autopsy is performed (Barrier et al. 2009).

In this study, osteometric analyses were carried out in addition to the 3D analyses. By placing easily identifiable points on the apex of the bone contours, we were able to obtain data in the traditional manner, which allowed us to verify that our data were in agreement with published values (Cavaignac et al. 2016). Origin-based variability (Purkait & Chandra 2004) must be taken into account in literature comparisons, but the results of these three reference measurements are consistent with published results (Cavaignac et al. 2016). Furthermore, the intra- and inter-observer error rates were very low in our study—none exceed 2%. These two aspects (reproducibility and plausibility) validate our methodology. If we had wanted to carry out an analysis based only on classic osteometric variables (EB, ADPMS, ADPLC), we would have had to consider the patients’ morphotype, hence their biometric data (height, weight, frontal plane morphotype, etc.). However, these variables (EC, ADPMS, ADPLC) were secondary outcome measures used to validate our measurement method by comparing it to existing data. We felt it was not necessary to weight these results with the biometric data, especially that our data were consistent with published values (Cavaignac et al. 2016). Geometric morphometric analysis eliminates differences related to object size.

The anatomical profiles of Asian knees are smaller and narrower than those of Caucasian knees (Yue et al. 2011). Most of the commercially available total knee arthroplasty (TKA) implants were designed based on anthropometric data of Caucasian knees, thus they may not be suitable for Asian patients (Bilfeld et al. 2012; Gonzalez et al. 2009; Ho et al. 2006; Yue et al. 2011). In a comparative study of the outcomes following TKA, Asian patients had significantly less postoperative range of motion and a higher rate of revision (Iorio et al. 2007). As the number of TKA procedures is expected to increase in Asia (Yang et al. 2012), it is essential to analyze the morphological characteristics of Asian knees to provide validated references for Asian TKA implants. We performed a shape-based analysis that removed size effects. This is a crucial issue for us, as the anatomical difference is not only related to differences in size. The simplistic solution that Chinese patients need smaller implants will only solve part of the problem. Not only do these implants need to be smaller, they need to have a different shape. Only the concept of anteroposterior length, mediolateral width and/or aspect ratio provide some insight into interethnic differences (Kim et al. 2017). Like Kim et al (Kim et al. 2017), we believe that these data create uncertainty around variability but do not answer the question itself.

Geometric morphometric analysis is a global 3D analysis that takes into account the location of each landmark in space relative to the others. Our analysis confirms that this dimorphism exists even when the size effect is removed. Furthermore, doing an analysis based on ratios or lengths over-simplifies the problem. It has been shown that soft tissue impingement due to overhang leads to postoperative pain and worse functional outcomes (Dai et al. 2014; Ho et al. 2006; Mahoney & Kinsey 2010). Reducing the size of the femoral component increases the risk of instability during knee flexion. If the femoral implant is shifted proximally to compensate for downsizing, the height of the joint line will be altered. For these reasons, only adjusting the size does not solve the problem – the shape must be taken into account.

The primary finding of our study is that ethnic dimorphism is present in the distal femur. The sex differences in distal femur from a Chinese population have been evaluated by Yang and colleagues (Yang et al. 2012). However, their study used classic osteometric methods and measured distances, angles and ratios in three dimensions without connecting these dimensions. In our study, the coordinates of each target point were analyzed in three dimensions and were related to the location of other points. Thus our study should be more properly called 3D analysis (Pretorius et al. 2006). It is also interesting to note that sexual dimorphism was more prevalent in the Asian population than the European one (Fig. 4). We chose to quantify sex-related differences in the context of both orthopedics and anthropology. The impact of gender is hotly debated in orthopedics; it appears that the size difference between men and women explains part of the differences (Bellemans et al. 2010). However, these differences are in part related to shape, independently of size (Fig. 4). Geometric morphometric analysis have revealed these shape-related differences. In the anthropology context, sex determination contributes to identifying human remains (Ozer & Katayama 2008; Purkait & Chandra 2004; Slaus et al. 2003).

The current study has certain limitations. Only skeletally mature subjects were included. In younger persons, the bone contours of the distal femoral epiphysis are not completely ossified. This would have increased the possibility of error during landmark placement by the observers. Moreover, diseases that do not affect the distal femur but may require a CT scan that includes the distal femur, such as vascular conditions, are more common in older subjects. We were not able to determine the number of subjects needed for this study, as this was the first time that morphometric geometry methods were used to analyze distal femur anatomy. We initially based our sample size calculation on data from the Yang study (Yang et al. 2014) (measuring BCB in an Asian population) and the Cavaignac study (Cavaignac et al. 2016) (measuring BCB in a European population). This calculation pointed to 35 subjects being needed in each group to reveal a difference of more than 4 mm between two ethnic groups using the BCB (common standard deviation of 6 mm, alpha risk of 0.05 and 90% power). But we felt it was timely to include a much larger number of subjects, making this the largest study to compare distal femur anatomy between two ethnic groups.

It is important to point out that our analysis of shape differences resulted in an average shape for each sub-group (Fig. 2). Although the average shapes differ, they do not capture all the variability within a population. The shape of Asian and European distal femurs differs, while the extremes of each group can have similar components. The APC circles in Figs. 3 and 4 are have some overlap become there are similarities between the populations. This is a drawback of “grouped” analysis, which suppresses individual characteristics. Most of the differences in shape in the orthopedic context occur in the axial plane (distal femoral twist, aspect ratio of distal femurs) (Kim et al. 2017; Mahfouz et al. 2012; Yip et al. 2004). We were somewhat surprised to found notable dimorphism in the frontal plane in our study (Fig. 2) – Asian femurs were longer from cranial to caudal than the European femurs. To our knowledge, this frontal dimorphism has never been shown. This may be one of the reasons why Asian TKA patients have worse range of motion results (Iorio et al. 2007).

This study is the first step in an effort to classify the variability in femur shape suggested by Mahfouz (Mahfouz et al. 2012) but in the three planes in space. We will add data from other ethnic groups to enrich our database.

The use of clinical investigations for anthropological purposes, after validation of the methods applied, also opens new fields for anthropology. The number of subjects who could be studied for anthropological purposes is greater than those in classic osteological collections.

## Conclusions

In summary, the distal femur exhibits ethnic and ethnic–sexual dimorphism. Three-dimensional geometric morphometric analysis made it possible to show these shape differences. The large number of subjects studied may help to modernize the references for certain bone measurements.

## References

[CR1] Barrier P, Dedouit F, Braga J, Joffre F, Rouge D, Rousseau H, Telmon N (2009). Age at death estimation using multislice computed tomography reconstructions of the posterior pelvis. J Forensic Sci.

[CR2] Bellemans J, Carpentier K, Vandenneucker H, Vanlauwe J, Victor J (2010). The John Insall Award: Both morphotype and gender influence the shape of the knee in patients undergoing TKA. Clin Orthop Relat Res.

[CR3] Bilfeld MF, Dedouit F, Rousseau H, Sans N, Braga J, Rouge D, Telmon N (2012). Human coxal bone sexual dimorphism and multislice computed tomography: geometric morphometric analysis of 65 adults. J Forensic Sci.

[CR4] Bilfeld MF, Dedouit F, Sans N, Rousseau H, Rouge D, Telmon N (2013). Ontogeny of size and shape sexual dimorphism in the ilium: a multislice computed tomography study by geometric morphometry. J Forensic Sci.

[CR5] Bookstein F (1978) the meaurment of biological shape and shape change. Berlin and New York: Springer-Verlag

[CR6] Cavaignac E, Savall F, Faruch M, Reina N, Chiron P, Telmon N (2016). Geometric morphometric analysis reveals sexual dimorphism in the distal femur. Forensic Sci Int.

[CR7] Cheng FB, Ji XF, Lai Y, Feng JC, Zheng WX, Sun YF, Fu YW, Li YQ (2009). Three dimensional morphometry of the knee to design the total knee arthroplasty for Chinese population. Knee.

[CR8] Dai Y, Scuderi GR, Penninger C, Bischoff JE, Rosenberg A (2014). Increased shape and size offerings of femoral components improve fit during total knee arthroplasty. Knee Surg Sports Traumatol Arthrosc.

[CR9] Elewa A (2010) Morphometrics for nonmorphometricians. Berlin and London; Springer.

[CR10] Gonzalez PN, Bernal V, Perez SI (2009). Geometric morphometric approach to sex estimation of human pelvis. Forensic Sci Int.

[CR11] Ho WP, Cheng CK, Liau JJ (2006). Morphometrical measurements of resected surface of femurs in Chinese knees: correlation to the sizing of current femoral implants. Knee.

[CR12] Iorio R, Kobayashi S, Healy WL, Cruz AI, Ayers ME (2007). Primary posterior cruciate-retaining total knee arthroplasty: a comparison of American and Japanese cohorts. J Surg Orthop Adv.

[CR13] Iscan MY, Shihai D (1995). Sexual dimorphism in the Chinese femur. Forensic Sci Int.

[CR14] Kim DI, Kim YS, Lee UY, Han SH (2013). Sex determination from calcaneus in Korean using discriminant analysis. Forensic Sci Int.

[CR15] Kim TK, Phillips M, Bhandari M, Watson J, Malhotra R (2017). What differences in morphologic features of the knee exist among patients of various races? A systematic review. Clin Orthop Relat Res.

[CR16] King CA, Iscan MY, Loth SR (1998). Metric and comparative analysis of sexual dimorphism in the Thai femur. J Forensic Sci.

[CR17] Klingenberg CP (2002). Morphometrics and the role of the phenotype in studies of the evolution of developmental mechanisms. Gene.

[CR18] Miriam Zelditch (2004) Geometrics morphometrics for biologists: a primer. Amsterdam and London; Elsevier

[CR19] Mahfouz M, Abdel Fatah EE, Bowers LS, Scuderi G (2012). Three-dimensional morphology of the knee reveals ethnic differences. Clin Orthop Relat Res.

[CR20] Mahoney OM, Kinsey T (2010). Overhang of the femoral component in total knee arthroplasty: risk factors and clinical consequences. J Bone Joint Surg Am.

[CR21] Mall G, Graw M, Gehring K, Hubig M (2000). Determination of sex from femora. Forensic Sci Int.

[CR22] Ozer I, Katayama K (2008). Sex determination using the femur in an ancient Japanese population. Coll Antropol.

[CR23] Pretorius E, Steyn M, Scholtz Y (2006). Investigation into the usability of geometric morphometric analysis in assessment of sexual dimorphism. Am J Phys Anthropol.

[CR24] Purkait R, Chandra H (2004). A study of sexual variation in Indian femur. Forensic Sci Int.

[CR25] Sheldon WH (1950). The somatotype, the morphophenotype and the morphogenotype. Cold Spring Harb Symp Quant Biol.

[CR26] Slaus M, Strinovic D, Skavic J, Petrovecki V (2003). Discriminant function sexing of fragmentary and complete femora: standards for contemporary Croatia. J Forensic Sci.

[CR27] Srivastava R, Saini V, Rai RK, Pandey S, Tripathi SK (2012). A study of sexual dimorphism in the femur among North Indians. J Forensic Sci.

[CR28] Trancho GJ, Robledo B, Lopez-Bueis I, Sanchez JA (1997). Sexual determination of the femur using discriminant functions. Analysis of a Spanish population of known sex and age. J Forensic Sci.

[CR29] Yang B, Yu JK, Gong X, Chen LX, Wang YJ, Wang J, Ma D (2012). Sex, age, and annual incidence of primary total knee arthroplasty: a university affiliated hospital survey of 3118 Chinese patients. Chin Med J (Engl).

[CR30] Yang B, Yu JK, Zheng ZZ, Lu ZH, Zhang JY (2014). Comparative study of sex differences in distal femur morphology in osteoarthritic knees in a Chinese population. PLoS One.

[CR31] Yip DK, Zhu YH, Chiu KY, Ng TP (2004). Distal rotational alignment of the Chinese femur and its relevance in total knee arthroplasty. J Arthroplasty.

[CR32] Yue B, Varadarajan KM, Ai S, Tang T, Rubash HE, Li G (2011). Differences of knee anthropometry between Chinese and white men and women. J Arthroplasty.

